# Fumitremorgin C Attenuates Osteoclast Formation and Function *via* Suppressing RANKL-Induced Signaling Pathways

**DOI:** 10.3389/fphar.2020.00238

**Published:** 2020-03-10

**Authors:** Yu Yuan, Kai Chen, Xi Chen, Chao Wang, Heng Qiu, Zhen Cao, Dezhi Song, Youqiang Sun, Jianmin Guo, Jennifer Tickner, Jiake Xu, Jun Zou

**Affiliations:** ^1^ School of Kinesiology, Shanghai University of Sport, Shanghai, China; ^2^ School of Biomedical Sciences, University of Western Australia, Perth, WA, Australia; ^3^ School of Physical Education and Sports Science, South China Normal University, Guangzhou, China; ^4^ Department of Orthopaedic Surgery, The First Affiliated Hospital of Wenzhou Medical University, Wenzhou, China; ^5^ School of Sports Science, Wenzhou Medical University, Wenzhou, China; ^6^ Department of Orthopedics, First Affiliated Hospital, Guangzhou University of Chinese Medicine, Guangzhou, China

**Keywords:** fumitremorgin C, osteoclast, osteoporosis, NFATc1, MAPK

## Abstract

Excessive bone resorption conducted by osteoclasts is considered as the main cause of osteoclast-related bone diseases such as osteoporosis. Therefore, the suppression of excessive osteoclast formation and function is one of the strategies to treat osteoclast-related bone diseases. Fumitremorgin C (Fum) is a mycotoxin extracted from *Aspergillus fumigatus*. It has been shown to have extensive pharmacological properties, but its role in the treatment of osteoclast-related bone diseases remains unclear. In this study, we aim to find out whether Fum can inhibit the receptor activator of nuclear factor-κB ligand (RANKL)-induced osteoclast formation and function. The results showed that Fum could significantly attenuate osteoclast formation and function at concentrations from 2.5 to 10 µM. The protein expression of bone resorption factors such as NFATc1, cathepsin K, V-ATPase-d2, and c-Fos was suppressed with the treatment of Fum at a concentration of 10 µM. In addition, Fum was also shown to suppress the activity of NF-κB, intracellular reactive oxygen species level, and MAPK pathway. Taken together, the present study showed that Fum could attenuate the formation and function of osteoclast *via* suppressing RANKL-induced signaling pathways, suggesting that Fum might be a potential novel drug in the treatment of osteoclast-related bone diseases.

## Introduction

Bone is a dynamic organ keeping constant remodeling throughout life. The dynamic balance of bone remodeling depends on the osteoblast-induced bone formation and the osteoclast-regulated bone resorption (He et al., 2013). Osteoclast is a key participant in regulating bone microstructure degeneration and bone loss, and excessive bone resorption is considered as the main cause of osteoclast-related bone diseases such as osteoporosis (Zhou et al., 2016).

Osteoclasts are multinucleated cells derived from hematopoietic cells of the monocyte/macrophage lineage and capable of resorbing mineralized bone matrix uniquely in the bone micro-environment (Chen et al., 2018). During osteoclastogenesis and bone resorption, both receptor activator of nuclear factor-κB ligand (RANKL) and M-CSF are the necessary cytokines for osteoclast formation and function (Karst et al., 2004). M-CSF plays an indispensable role in the survival, formation, and function of osteoclast (De Vries et al., 2015). RANKL, a TNF ligand superfamily member, is an essential cytokine during osteoclastogenesis and bone resorption *via* interacting with RANK (Kreja et al., 2007). RANK is essential for osteoclast survival, formation, and activation as well (Dougall, 2012). The interaction between RANKL and RANK leads to the activation of NF-κB, MAPK, and NFATc1 signaling (Song et al., 2016).

Fumitremorgin C (Fum), the molecular formula of which is C_22_H_25_N_3_O_3_, is a mycotoxin extracted from *Aspergillus fumigatus*. It has been shown to have a wide range of pharmacological properties such as reversing drug resistance to mitoxantrone, doxorubicin, and topotecan which are common antitumor drugs (Rabindran et al., 1998; Gonzalez-Lobato et al., 2010). Nevertheless, the cellular and molecular actions of Fum on osteoclast formation and function still remain unknown. In this study, we found that Fum was capable of inhibiting osteoclastogenesis based on the results of our initial compound screening. Thus, we aim to assess the impacts of Fum on RANKL-induced osteoclast formation and bone resorption and to investigate the mechanism whereby Fum affects the formation and function of osteoclast. We found that Fum could attenuate osteoclastogenesis and bone resorption *via* attenuating RANKL-induced signaling pathways, suggesting that Fum might be one of the potential novel drug candidates for osteoclast-related bone diseases.

## Materials and Methods

### Materials and Reagents

Fum, with purity >99%, was kindly provided by Prof. Renxiang Tan's lab (Nanjing University, China) (Ma et al., 2006). Fum was dissolved in dimethyl sulfoxide (DMSO) and the concentration of the stock solution was 100 mM. It was further diluted to the concentration for intervention with cell culture medium. The same concentration of DMSO (0.01% final concentration) was assigned as vehicle control. Fetal bovine serum (FBS) and αMEM were obtained from Thermo Fisher Scientific (Scoresby, Australia). The MTS and luciferase reporter assay kits were obtained from Promega (Sydney, Australia). The primary antibodies used for β-actin, p38, phospho-p38, NFATc1, IκB-α, cathepsin K, ERK, and phospho-ERK were obtained from Santa Cruz Biotechnology (Dallas, USA). The primary antibodies used for c-Fos, phospho-JNK, and JNK were obtained from Cell Signaling Technology (Danvers, USA). Fluo-4 calcium flux indicator, 2',7'-dichlorodihydrofluorescein diacetate (H2DCFDA), and rhodamine phalloidin were purchased from Thermo Fisher Scientific. DAPI was obtained from Santa Cruz. The recombinant GST-rRANKL protein and M-CSF were produced and used as described previously (Xu et al., 2000; Liu et al., 2013).

### *In Vitro* Osteoclastogenesis Assay

Bone marrow-derived macrophages (BMMs) were isolated from 6-week-old C57BL/6 mice and then cultured in the complete αMEM medium (10% FBS, 1% penicillin and streptomycin, and 89% αMEM) containing M-CSF (50 ng/ml). BMMs were seeded in a 96-well plate at a density of 6 × 10^3^ cells/well and cultured in the complete αMEM medium containing M-CSF after 72 h. BMMs were stimulated with GST-rRANKL (50 ng/ml) with or without range concentrations of Fum (1, 2.5, 5, and 10 µM) on the following day. The medium was changed every 48 h until osteoclasts formed. After 5 days of culture, phosphate-buffered saline (PBS) was used to wash the cells and then 2.5% glutaraldehyde solution was used to fix the cells for 10 min. The cells were then stained for TRAcP activity using a leucocyte acid phosphatase staining kit (Sigma-Aldrich, Australia) as described previously (Chen et al., 2019). The TRAcP-positive cells with at least three nuclei were calculated as osteoclasts. The protocol was approved by the University of Western Australia Animal Ethics Committee (RA/3/100/1244).

### Cytotoxicity Assay

The cytotoxic effects of Fum on osteoclast precursors were examined using an MTS assay kit (Promega, Australia). BMMs were seeded into a 96-well plate at a density of 6 × 10^3^ cells/well and cultured in the complete αMEM medium overnight. After that, the cells were incubated in the presence of Fum at various concentrations (0, 0.25, 0.5, 1, 2.5, 5, and 10 µM) for 48 h. Then, the cells were treated with MTS solution for 120 min. The absorbance of MTS was detected at 490 nm by spectrophotometric absorbance *via* a microplate reader (BMG, Germany).

### Staining for Actin Belt Formation

BMMs were induced to mature osteoclasts as stated above. After that, the mature osteoclasts were washed two to three times with PBS, and then 4% paraformaldehyde was used to fix the osteoclasts for 10 min. Subsequently, Triton X-100 (0.1%) was used to permeabilize the osteoclasts and then the osteoclasts were blocked in BSA (3%). After that, rhodamine phalloidin was diluted in PBS at a volume ratio of 1:300 and used to stain the osteoclasts. Then, the osteoclasts were incubated and protected from light for 120 min. The osteoclasts were incubated with DAPI for 10 min. Subsequently, NIKON A1Si confocal microscopy was used to observe the osteoclasts after the treatment with ProLong Gold antifade mountant. The images from each group were captured randomly and the percentage of actin belts area was measured *via* ImageJ.

### Hydroxyapatite Resorption Assay

The effects of Fum on the function of osteoclasts was determined *via* hydroxyapatite resorption assay as previously described (Chen et al., 2018). The BMMs were plated in a six-well collagen-coated culture plate at a density of 1 × 10^5^ cells/well and cultured in the complete αMEM medium containing 50 ng/ml M-CSF and RANKL. The cells were collected using a cell dissociaton solution as soon as the osteoclasts formed. After that, the cells were plated in a 96-well hydroxyapatite-coated plate (Corning, USA) and cultured in the complete αMEM medium supplemented with M-CSF and RANKL, in the absence or presence of Fum (0, 2.5, and 10 μM). In each group, one-half of the wells were used for TRAcP staining to calculate the number of osteoclasts, and the other wells were bleached and then dried for capturing hydroxyapatite resorption visualization *via* a Nikon microscope. Image J was used to analyze the percentage of resorption area.

### Determination of Intracellular ROS Production

After the stimulation with RANKL with or without Fum (0, 2.5, and 10 μM), the intracellular ROS production of BMMs was detected according to Lee's protocol (Lee et al., 2005). The cells were washed and cultured in Hank's balanced salt solution including 5 mM H2DCFDA for 60 min. H2DCFDA is oxidated and subsequently transformed into 2′,7′-dichlorofluorescein (DCF) which is highly fluorescent. By using an A1Si confocal microscope (NIKON), the fluorescence intensity of DCF was detected under an optimized excitation/emission wavelength at 488/515–540 nm. The percentage of ROS-positive cells and the mean fluorescence intensity were measured *via* Image J.

### NF-κB or NFATc1 Luciferase Assays

In order to examine the activation of NF-κB and NFATc1, an NF-κB luciferase reporter gene and an NFATc1 luciferase reporter gene (Wang et al., 2003; Cheng et al., 2018) were stably transfected into the RAW264.7 cells, respectively, as previously described. Then, the RAW264.7 cells were seeded into a 48-well plate, respectively, to allow attachment. The density of the RAW264.7 cells with an NF-κB luciferase reporter gene was 1.5 × 10^5^ cells per well, and the density of the RAW264.7 cells with an NFATc1 luciferase reporter gene was 5 × 10^4^ cells per well. After that, the RAW264.7 cells were pre-treated for 60 min with Fum until 80% confluent and then treated with RANKL (50 ng/ml) for 6 and 24 hours, respectively. After stimulation, the RAW264.7 cells were collected for detecting luciferase activity with a luciferase reporter gene assay substrate (Promega, Australia) *via* a BMG Polar Star Optima luminescence reader (Germany).

### Western Blot Assay

For Western blot (WB) assay, the BMMs were seeded into a six-well plate at a density of 1.0 × 10^5^ cells/well and then cultured in the complete medium including M-CSF and RANKL with or without Fum(10 μM) as previously described (Chen et al., 2018). After the intervention, the cells were lysed to harvest protein using the radioimmunoprecipitation lysis buffer. SDS-polyacrylamide gel electrophoresis (SDS-PAGE) was used to separate the total protein. The sum of 30 µg total protein was loaded onto 8% SDS-PAGE gels and then was transferred to the nitrocellulose membrane (GE Healthcare, Australia). Tris-glycine buffer was used as a transfer buffer in this experiment. After that, the membranes were soaked with 5% skimmed milk [diluted in Tris-HCl-buffered saline supplemented with 0.1% Tween-20 (TBST)] and then incubated with primary antibodies (diluted in 1% skimmed milk at 1:1,000) under gentle shaking at 4°C overnight. The membranes were washed with TBST. Then, horseradish peroxidase-conjugated secondary antibody was used to incubate the membranes for 60 min at room temperature the following day. After that, the membranes were analyzed using enhanced chemiluminescence reagents *via* Image quant LAS 4000 (GE Healthcare, Australia). The intensity of the bands on the membranes was analyzed using ImageJ software.

### Statistical Analysis

The experiments were independently repeated three times or more. All data were expressed as mean ± standard deviation (SD). One-way ANOVA test was used to compare the means of all the groups, followed by Bonferroni's *post hoc* test. Student's t-test was used for comparisons between two groups, and *p* < 0.05 was considered to be statistically significant.

## Results

### Fum Suppresses RANKL-Induced Osteoclastogenesis

**Figure 1:**
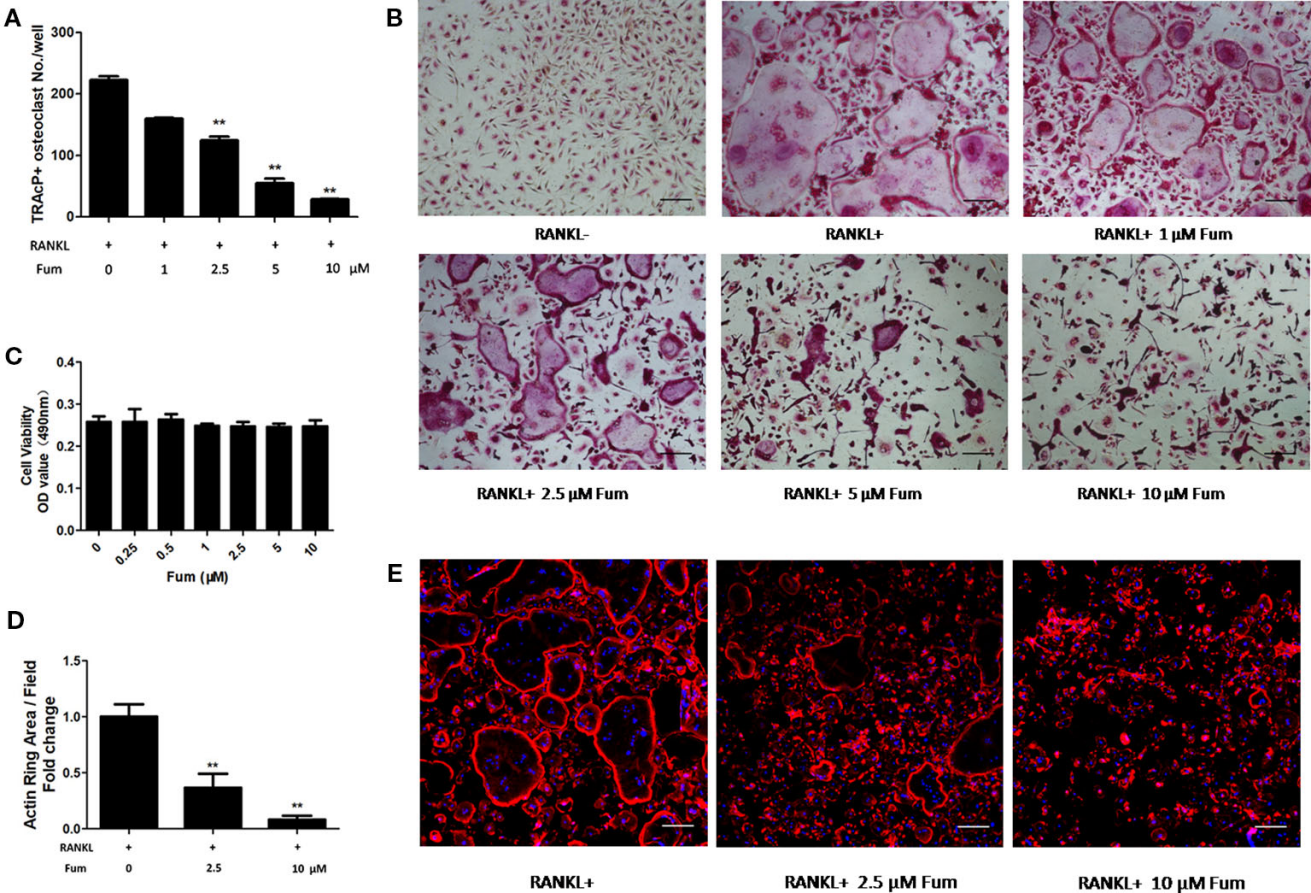
Fum attenuates RANKL-induced osteoclastogenesis. **(A)** Quantification of TRAcP-positive osteoclasts under varying concentrations of Fum (*n* = 3). **p < 0.01 relative to the Fum-untreated control. **(B)** Representative images of osteoclasts under the treatment of different concentrations of Fum for 5 days. **(C)** Proliferation of BMMs treated with Fum at varying concentrations for 48 h was detected using the MTS assay (*n* = 3). **(D)** The average area of the actin belt was measured *via* Image-J (*n* = 3). **p < 0.01 relative to the Fum-untreated control. **(E)** Representative images of the actin belt of osteoclasts were captured *via* confocal microscopy. Scale bar = 100 µm. Fum, fumitremorgin C; TRAcP, tartrate-resistant acid phosphatase; BMMs, bone marrow macrophages; RANKL, receptor activator of nuclear factor-κB ligand.

**Figure 2:**
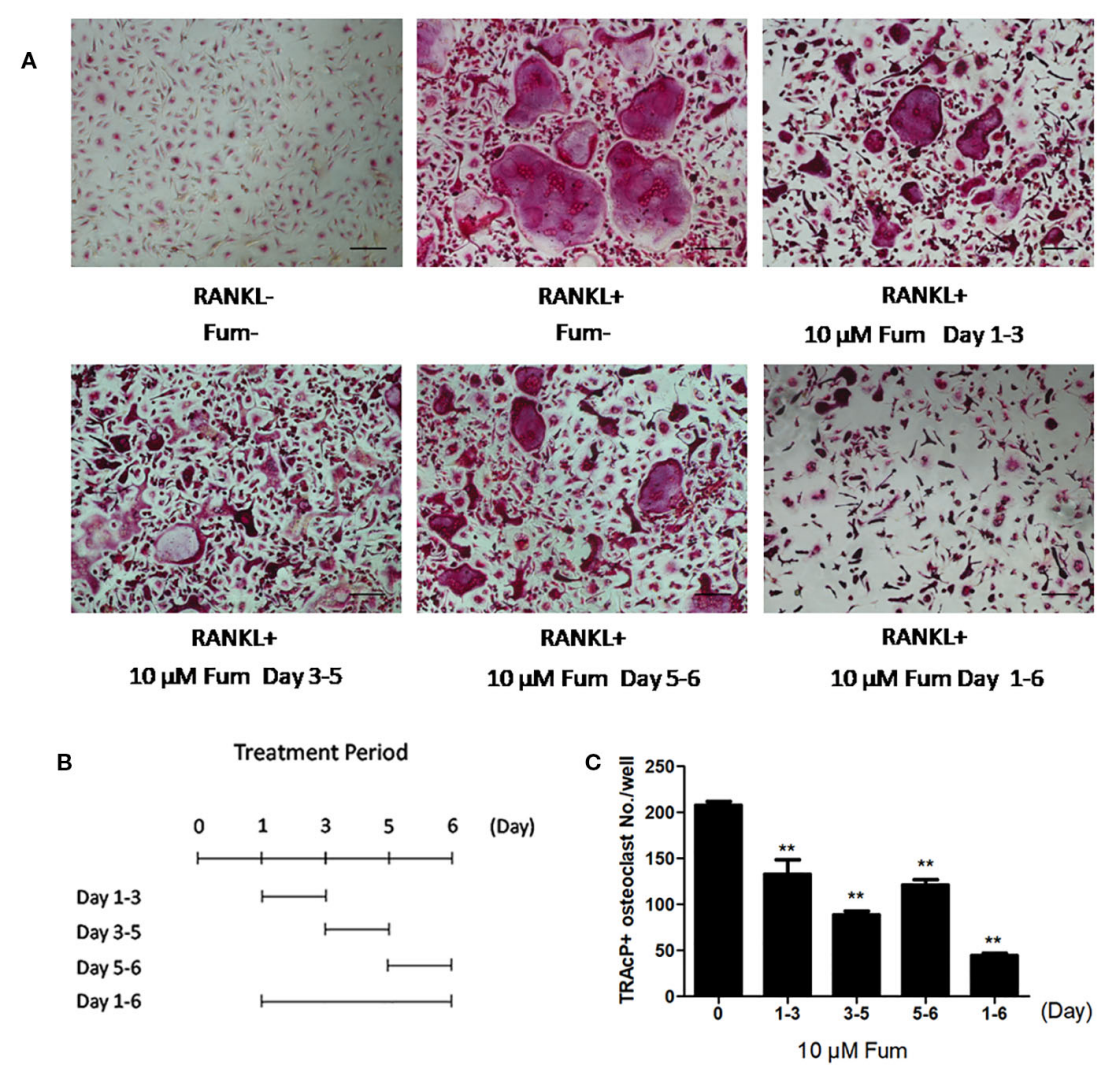
Fum principally inhibits RANKL-induced osteoclast formation at the middle stage. **(A)** Representative images of osteoclasts under the treatment of 10 μM Fum in different stages during the process of osteoclastogenesis. **(B)** The time periods in the presence of Fum. **(C)** The number of TRAcP-positive osteoclasts under the treatment of Fum was counted (*n* = 3). **p < 0.01 relative to the Fum-untreated control. Scale bar = 100 μm. Fum, fumitremorgin C; RANKL, receptor activator of nuclear factor-κB ligand; TRAcP, tartrate-resistant acid phosphatase.

To detect the effects of Fum on osteoclastogenesis, BMMs were seeded in a 96-well plate at 6 × 10^3^/well and treated with RANKL under varying concentrations of Fum. The results showed that Fum was capable of suppressing osteoclastogenesis in a dose-dependent manner (**Figures 1A, B**). The number of TRAcP-positive osteoclasts was obviously decreased at 2.5 µM Fum and higher concentrations. MTS assay was performed to detect any cytotoxic effects of Fum on BMMs. The MTS assay results indicated that there was no cytotoxic effects of Fum on BMMs at different concentrations in the present research (**Figure 1C**). To determine the effects of Fum on the formation of F-actin belt which is essential for osteoclast function, rhodamine phalloidin was used to stain the osteoclasts so as to observe the morphological structure of F-actin. Fum was shown to inhibit F-actin belt formation and reduce the actin belts area significantly at the concentration of 2.5 µM Fum and higher (**Figures 1D, E**). We also observed the effects of Fum on the different stages of osteoclastogenesis and found that 10 µM Fum could inhibit osteoclast formation in different stages during the process of osteoclastogenesis, specially in the middle stage (days 3–5) (**Figure 2**). All of these results suggest that Fum is capable of inhibiting RANKL-induced osteoclastogenesis without obvious cytotoxicity on BMMs.

### Fum Attenuates RANKL-Induced Osteoclast Resorptive Function

**Figure 3:**
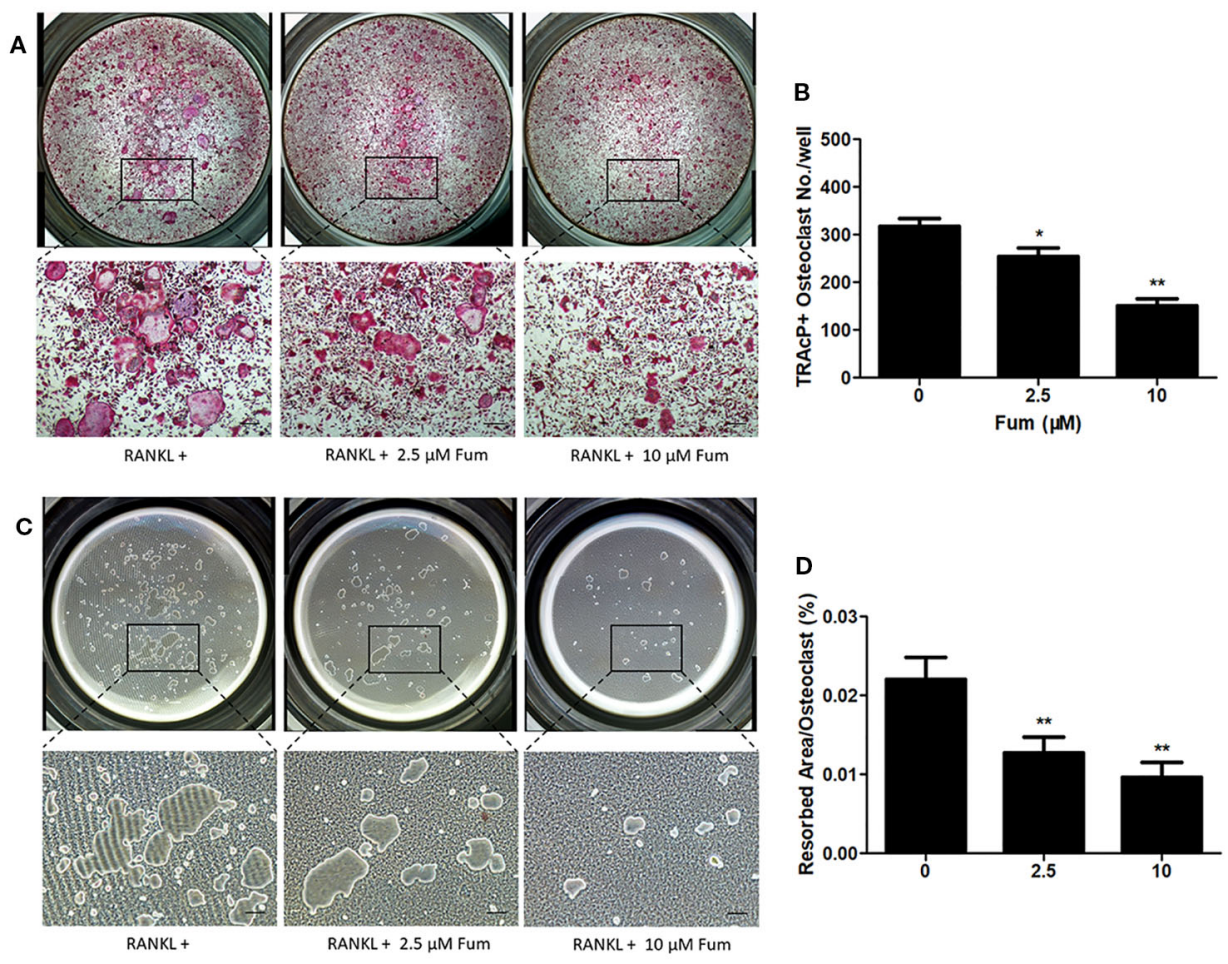
Fum suppresses the resorptive function of osteoclast. **(A)** Representative images of TRAcP-positive multinucleated osteoclasts under the treatment of Fum in the hydroxyapatite-coated plates. **(B)** The number of TRAcP-positive osteoclasts under the treatment of Fum. *n* = 3. *p < 0.05, **p < 0.01 relative to the Fum-untreated control. **(C)** Representative images of osteoclast resorptive function on hydroxyapatite-coated surfaces. **(D)** The percentage of osteoclast resorptive area on hydroxyapatite-coated surfaces. *n* = 3. **p < 0.01 relative to the Fum-untreated control. Scale bars = 100 μm. Fum, fumitremorgin C; RANKL, receptor activator of nuclear factor-κB ligand; TRAcP, tartrate-resistant acid phosphatase.

In order to detect the effects of Fum on the resorptive function of osteoclasts, hydroxyapatite-coated plates were used to culture the mature osteoclast in the presence of Fum. The results showed that both the number of multinucleate osteoclasts and the percentage of osteoclast-resorbed area were decreased obviously in the presence of Fum (**Figures 3A–C**). The percentage of osteoclast resorptive area was decreased obviously as well (**Figure 3D**). All of these results together demonstrated that Fum could attenuate the RANKL-induced resorptive function of osteoclasts.

### Fum Suppresses RANKL-Induced ROS Generation

**Figure 4:**
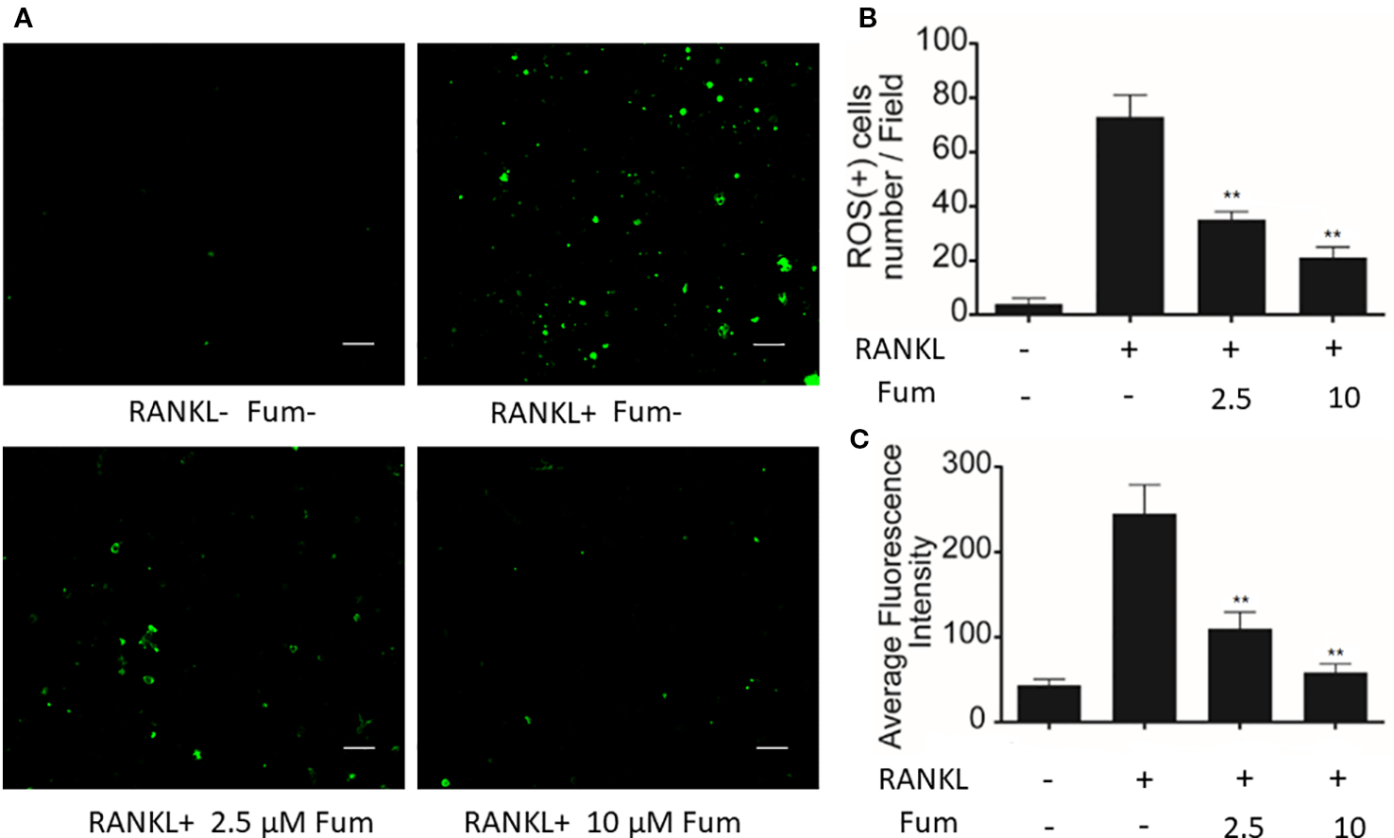
Fum suppresses ROS generation induced by RANKL. **(A)** Representative images of ROS generation in BMMs in the absence or presence of Fum. Scale bar = 100 μm. **(B)** Quantification of ROS-positive cells per field (*n* = 3). **(C)** Quantitative analyses of fluorescence intensity averaged on osteoclast precursors (*n* = 3). **p < 0.01 relative to the Fum-untreated control. Fum, fumitremorgin C; ROS, reactive oxygen species; RANKL, receptor activator of nuclear factor-κB ligand; TRAcP, tartrate-resistant acid phosphatase.

In order to detect the effects of Fum on RANKL-induced ROS generation in BMMs, H2DCFDA, commonly known as a sensitive probe for ROS, was used to test intracellular ROS generation *via* confocal microscopy. The results showed that Fum was capable of reducing ROS-positive cells and inhibiting the intensity of DCF fluorescence significantly in BMMs under the treatment of RANKL in a dose-dependent manner (**Figure 4**), which suggested that Fum could suppress RANKL-induced ROS generation in osteoclast precursors.

### Fum Interferes With RANKL-Induced NF-κB Activity and MAPK Signaling

**Figure 5:**
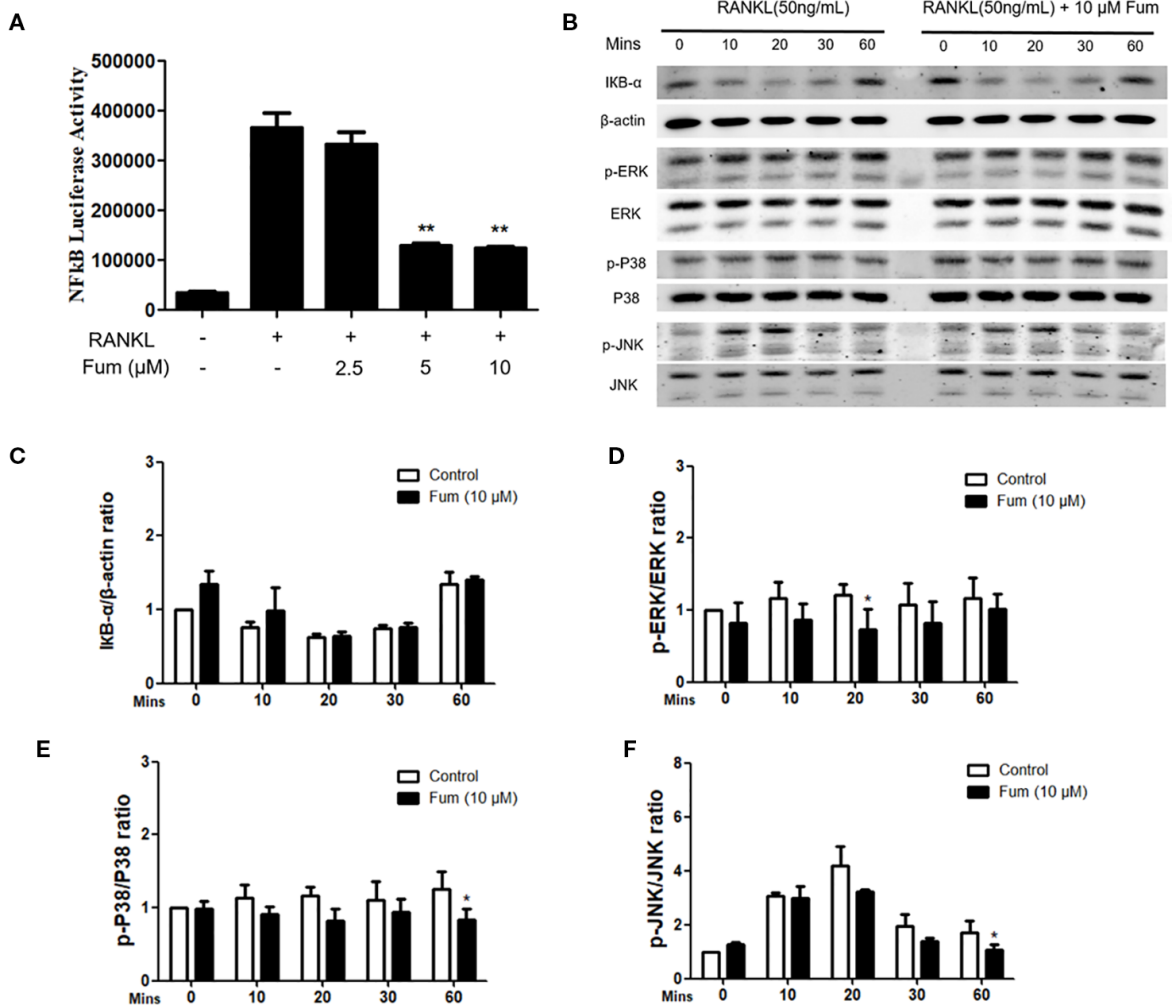
Fum attenuates osteoclast formation through inhibiting NF-κB activity and MAPK signaling. **(A)** The results of NF-κB luciferase reporter assay showed that Fum significantly suppressed NF-κB activity at the concentrations of 5 µM Fum and higher. The luciferase activity of NF-κB was detected by a BMG luminescence reader (*n* = 3). **p < 0.01 relative to the Fum-untreated control. **(B)** Representative WB images of the inhibiting effects of Fum on MAPK signaling pathways including P38, ERK, and JNK. BMMs were pretreated with Fum at 10 μM for 60 min and then cultured in the complete medium containing RANKL for 0, 10, 20, 30, and 60 min. The total protein was extracted after intervention. **(C)** The ratios of IκBα band intensity relative to the β-actin bands. *n* = 3. **(D**–**F)** The ratios of p-P38, p-ERK, and p-JNK band intensity relative to total P38, ERK, and JNK bands (*n* = 3). The intensity of the bands were analyzed using Image-J. *p < 0.05, **p < 0.01 relative to the Fum-untreated control. Fum, fumitremorgin C; RANKL, receptor activator of nuclear factor-κB ligand; NF-κB, nuclear factor-κB; MAPK, mitogen-activated protein kinases; BMMs, bone marrow macrophages; WB, western blot; ERK, extracellular signal-regulated kinase; JNK, c-Jun N-terminal kinase.

In order to understand the mechanism whereby Fum inhibits osteoclast formation, NF-κB luciferase assay and WB assay were performed. An NF-κB luciferase reporter was stably transfected into the RAW264.7 cells, and then the RAW264.7 cells were pretreated with different concentration gradients of Fum for 60 min and then cultured in the complete medium containing RANKL for another 6 h. The results of the NF-κB luciferase reporter assay showed that Fum could significantly inhibit the activity of NF-κB at the concentrations of 5 µM Fum and higher (**Figure 5A**). For WB assay, BMMs were pretreated with Fum and stimulated with RANKL for 0, 10, 20, 30, and 60 min. The WB results showed that Fum had no significant effects on the degradation of IκBα, but it could attenuate the phosphorylation of ERK, p38, and JNK (**Figures 5B–F**), indicating that Fum had inhibiting effects on RANKL-induced MAPK signaling pathways.

### Fum Suppresses RANKL-Induced NFATc1 Activation

**Figure 6:**
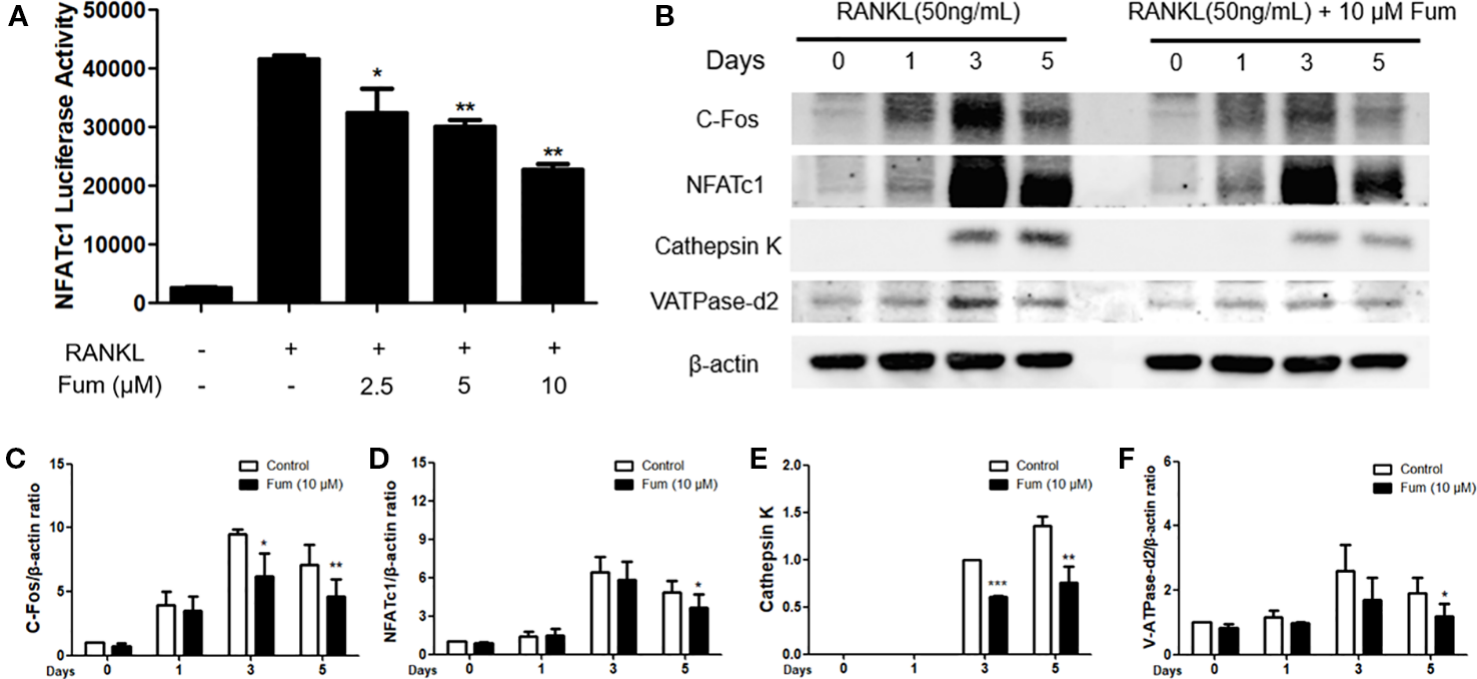
Fum suppresses NFATc1 activity, the upstream and downstream proteins of NFATc1. **(A)** The results of the NFATc1 luciferase assay showed that Fum obviously inhibited the activity of NFATc1 dose-dependently. The luciferase activity of NFATc1 was detected by a BMG luminescence reader (*n* = 3). *p < 0.05, **p < 0.01 relative to the Fum-untreated control. **(B)** Representative WB images of NFATc1 and its upstream and downstream proteins containing cathepsin K, c-Fos, and V-ATPase-d2. **(C**–**F)** The ratios of the band intensity of NFATc1, c-Fos, V-ATPase-d2, and cathepsin K relative to β-actin (*n* = 3). The intensity of the bands was analyzed through Image-J. *p < 0.05, **p < 0.01, ***p < 0.001 relative to the Fum-untreated control. Fum, fumitremorgin C; NFATc1, nuclear factor of activated T cells 1; RANKL, receptor activator of nuclear factor-κB ligand; WB, western blot.

NFATc1 luciferase reporter assay and WB assay were performed to examine the effects of Fum on NFATc1 activity and on downstream factors. For luciferase reporter assay, an NFATc1 luciferase reporter was stably transfected into the RAW264.7 cells and then the RAW264.7 cells were pretreated with different concentration gradients of Fum for 60 min and then cultured in the complete medium supplemented with RANKL for another 24 h. The results of the NFATc1 luciferase assay showed that Fum could obviously inhibit the activity of NFATc1 at 2.5 µM Fum and at higher concentrations (**Figure 6A**). For the WB assay, BMMs were cultured in complete medium supplemented with RANKL for 0, 1, 3, and 5 days with or without the treatment of Fum. The WB results also showed that Fum could inhibit the expression of NFATc1 during osteoclast differentiation (**Figure 6B**). The upstream protein (c-Fos) and downstream proteins (cathepsin K and V-ATPase-d2) of NFATc1 were shown to be down-regulated in the presence of Fum. All of these data together indicated that Fum (**Figures 6C–F**) could suppress RANKL-induced NFATc1 activation effectively.

## Discussion

The balance of bone remodeling depends on bone-resorbing osteoclasts and bone-forming osteoblasts. Osteoclasts play an essential role in regulating bone remodeling, and excessive bone resorption regulated by osteoclasts is considered as the main cause of osteoclast-related bone diseases (Sun et al., 2019). Thus, the inhibition of excessive osteoclast formation and function is an effective method in the prevention and treatment of osteoclast-related bone diseases such as osteoporosis.

Fum, known as a potent ABCG2/breast cancer resistance protein (BCRP) inhibitor that reverses multidrug resistance, is a mycotoxin extracted from *Aspergillus fumigatus*, which has been shown to have a wide range of pharmacological properties (Rabindran et al., 2000; Liu et al., 2006; Gonzalez-Lobato et al., 2010). However, whether Fum can affect osteoclasts formation and function remains unclear. In the present research, we examined the effects of Fums on the formation and the function of osteoclasts. These results showed that Fum was capable of inhibiting RANKL-induced formation and function of osteoclasts. It is the first time that Fum was shown to attenuate osteoclastogenesis and bone resorption through attenuating RANKL-induced signaling pathways. The results indicated that Fum significantly suppressed RANKL-induced osteoclastogenesis, especially at middle stages, in a dose-dependent manner without cytotoxicity. Fum was shown to inhibit bone resorption effectively as well, suggesting that Fum might be a potential drug for osteoclast-related bone diseases such as osteoporosis. To investigate the mechanism whereby Fum inhibited osteoclastogenesis, we detected the activity of NF-κB using luciferase assay and discovered that Fum could suppress the activity of NF-κB significantly. NF-κB has been reported to play a key role in osteoclast formation. IκB-α would be degraded under the stimulation of RANKL and thus lead to NF-κB activation (Mercurio and Manning, 1999; Zhou et al., 2016). However, in the present study, Fum had a tendency of attenuating IκB-α protein degradation but not significantly, which suggested that Fum had inhibitory effects on NF-κB activity independent of IκB-α degradation.

NFATc1 is an essential signaling pathway leading to osteoclastogenesis as well (Charles et al., 2012). It is a master transcriptional regulator during the terminal differentiation of osteoclast. NFATc1 up-regulates in BMM under the stimulation of RANKL and reaches the peak value after 72 h. NFATc1 maintains strong expression in an autoamplification *via* binding to its own promoter (Asagiri et al., 2005). After reaching the peak value, the expression of NFATc1 begins to down-regulate due to NFATc1 ubiquitination and subsequent proteasomal degradation of NFATc1 in the late stage of osteoclastogenesis (Kim et al., 2010; Narahara et al., 2019). In the present study, the protein level of NFATc1 was shown to increase in the presence of RANKL, reach the peak value on day 3, and decrease on day 5, which was in concordance with the above studies. The results of the luciferase reporter assay showed that Fum was capable of suppressing the activity of NFATc1 during osteoclast differentiation. Furthermore, the WB results indicated that Fum down-regulated the protein expression of NFATc1 during osteoclastogenesis induced by RANKL as well. These results were in concordance with the inhibiting effects of Fum on osteoclast formation and function. Taken together, Fum might attenuate osteoclastogenesis and bone resorption *via* inhibiting the activation of NFATc1. In order to further investigate the effects of Fum on the NFATc1 signaling pathway, we also detected the protein expression of bone resorption factors including c-Fos, cathepsin K, and V-ATPase-d2 using Western blot analysis.

C-Fos, a direct transcriptional regulator of NFATc1, is critical for osteoclast formation (Wagner and Eferl, 2005). During osteoclastogenesis, c-Fos increases under the stimulation of RANKL and binds to the promoter region of NFATc1, leading to the expression of NFATc1 (Kim et al., 2013). There is a synergistic effect between c-Fos and NFATc1, which induces the expression of key bone resorption factors such as cathepsin K and V-ATPase-d2 (Matsumoto et al., 2004; Lee et al., 2006).

Cathepsin K, a downstream protein of NFATc1 signaling pathway, is essential for bone resorption and bone remodeling. Gene mutations of cathepsin K in humans might exhibit skeletal abnormalities such as frequent pathological fracture, short stature, and osteolysis (Pang et al., 2019). Cathepsin K-deficient mice were shown to cause the hypermineralization of the long bone and growth plates and develop osteopetrosis (Kiviranta et al., 2005; Boskey et al., 2009).

With the treatment of M-CSF and RANKL, osteoclast precursors fuse to become mature and functional osteoclasts during osteoclastogenesis. V-ATPase-d2 is an essential regulator in the process of osteoclast cell–cell fusion (Oh et al., 2015a). It was reported that the V-ATPase-d2 deficient mice exhibited impaired osteoclast fusion and enhanced bone formation (Lee et al., 2006). Consistent with the inhibiting effects of Fum on NFATc1 activation, Fum was shown to suppress the protein expression of c-Fos, cathepsin K, and V-ATPase-d2, which further indicated that Fum could suppress osteoclast formation and function through suppressing the NFATc1 signaling pathway.

During the process of osteoclastogenesis induced by RANKL, the MAPK pathways including ERKs, p38, and JNKs play critical roles as well (Lee et al., 2016). The inhibition of ERKs was reported to suppress the proliferation and fusion of osteoclast precursors (Oh et al., 2015b). JNK is an important regulator in the process of osteoclast formation, complete blockade of which will attenuate osteoclastogenesis (Vaira et al., 2008). It has been reported that p38 is crucially involved in osteoclastogenesis and capable of enhancing osteoclast maturation and function (Li et al., 2003; He et al., 2012). In the present study, Fum was shown to suppress the phosphorylation of ERKs, p38, and JNKs, which was consistent with the inhibiting effects of Fum on osteoclastogenesis and bone resorption.

In addition, reactive oxygen species (ROS) are also highly correlated with the formation and function of osteoclasts (Lee et al., 2005; Yip et al., 2005). It has been reported that the decrease of intracellular ROS production could attenuate osteoclastogenesis and bone resorption. ROS were reported to be involved in NFATc1 induction (Ke et al., 2006; Ghosh et al., 2018) and active MAPKs pathways as a physiologic second messenger (Kamata et al., 2005), thus further enhancing the formation and function of osteoclasts. Therefore, we detected the effects of Fum on ROS generation in the present study and discovered that Fum could suppress RANKL-induced ROS generation significantly in BMMs, suggesting that Fum might attenuate the NFATc1 and MAPKs pathways *via* acting as a ROS inhibitor.

**Figure 7:**
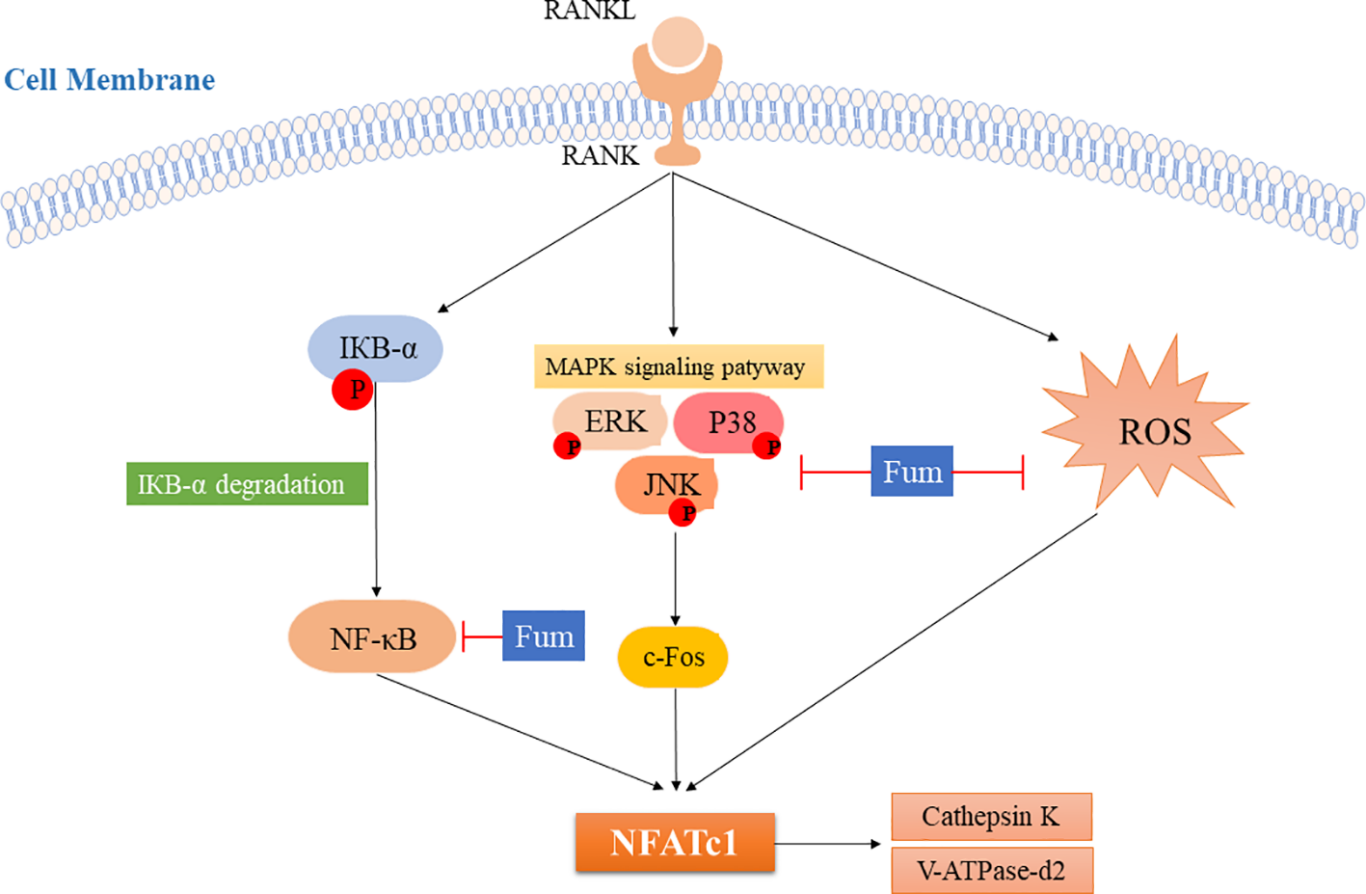
The model for inhibiting the effects of Fum on RANKL-induced NFATc1 activation during osteoclastogenesis. ROS production and the NF-κB and MAPKs pathways are activated after RANKL binding to RANK, leading to the increase of c-Fos and the activation of NFATc1. As a result, the bone resorption molecules, including cathepsin K and V-ATPase-d2, are up-regulated. The present study showed for the first time that Fum attenuates RANKL-induced osteoclastogenesis and bone resorption *via* inhibiting the activation of NFATc1, indicating a mechanism for inhibiting the effects of Fum on RANKL-induced osteoclasts formation and function. Fum, fumitremorgin C; ROS, reactive oxygen species; RANKL, receptor activator of nuclear factor-κB ligand; NFATc1, nuclear factor of activated T cells 1; NF-κB, nuclear factor-κB; MAPK, mitogen-activated protein kinases; ERK, extracellular signal-regulated kinase; JNK, c-Jun N-terminal kinase.

In summary, Fum could attenuate RANKL-induced osteoclastogenesis and bone resorption *via* inhibiting the activation of NFATc1 (**Figure 7**). The present study showed a mechanism whereby Fum attenuates RANKL-induced osteoclasts formation and function. Fum might be a potential novel drug in the treatment of osteoclast-related bone diseases due to its potent inhibitory effects on osteoclastogenesis and bone resorption.

## Data Availability Statement

All datasets generated for this study are included in the article.

## Author Contributions

YY, KC, XC, CW, and HQ performed the cell culture, the hydroxyapatite resorption assay, and the Western blot analysis. ZC, DS, YS, and JG performed the MTS assay, the luciferase assays, and the TRAcP staining. YY, KC, and JT wrote the manuscript. JX and JZ designed the overall study, supervised the project, and revised the manuscript.

## Funding

The present study was supported by grants from the Australian Health and Medical Research Council (NHMRC, Nos. 1107828, 1127156, and 1163933). The study was also supported by the China Postdoctoral Science Foundation funded project (Nos. 2018M640792 and 2019T120739), the Shanghai Key Lab of Human Sport Competence Development and Maintenance (Shanghai University of Sport) (No. 11DZ2261100), and the National Natural Science Foundation of China (No. 81572242).

## Conflict of Interest

The authors declare that the research was conducted in the absence of any commercial or financial relationships that could be construed as a potential conflict of interest.
